# Knowledge and attitude about disabilities in leprosy: Effects of an intervention grounded on the Meaningful Learning Theory

**DOI:** 10.1590/1980-220X-REEUSP-2021-0474

**Published:** 2022-02-04

**Authors:** Emanuelle Malzac Freire de Santana, Karen Krystine Gonçalves de Brito, Matheus de Medeiros Nóbrega, Ester Missias Villaverde Antas, Alana Tamar Oliveira de Sousa, Simone Helena dos Santos Oliveira

**Affiliations:** 1 Faculdades Nova Esperança, Departamento de Fisioterapia, João Pessoa, PB, Brazil.; 2 Faculdades Nova Esperança, Departamento de Enfermagem, João Pessoa, PB, Brazil.; 3 Universidade Federal da Paraíba, Programa de Pós-graduação em Enfermagem, João Pessoa, PB, Brazil.; 4 Universidade Federal da Campina Grande, Unidade Acadêmica de Enfermagem, Campina Grande, PB, Brazil.

**Keywords:** Leprosy, Knowledge, Attitude, Disabled Persons, Primary Health Care, Lepra, Conocimiento, Actitud, Personas con Discapacidad, Atención Primaria de la Salud, Hanseníase, Conhecimento, Atitude, Pessoas com Deficiência, Atenção Primária à Saúde

## Abstract

**Objective:**

To analyze the effects of an educational intervention in the light of the Meaningful Learning Theory on the knowledge and attitude of Primary Health Care physicians and nurses in the assessment of the degree of physical disability in leprosy.

**Method:**

An intervention study of the before-and-after type, conducted with 122 professionals (84 nurses and 38 physicians) from the Primary Health Care of João Pessoa, Paraíba, in a training course on the assessment of the degree of physical disability in leprosy. The data were collected with the research’s own instrument validated and analyzed by the chi-square adherence and proportion test, with a 5% significance level.

**Results:**

There was an increase in the scores of all items of the instrument, with a statistically significant difference (p < 0.05) in 20 of the 32 items, with emphasis on those related to the professional’s technical ability to conduct the stages of anamnesis, palpation of peripheral nerves, sensory and motor evaluation. It is also noteworthy that, after the intervention, 5 items obtained 100% of correct answers.

**Conclusion:**

The educational intervention grounded on the Meaningful Learning Theory improved the health professionals’ knowledge and attitude in the assessment of the degree of physical disability in people with leprosy.

## RESUMO

**Objetivo:**

Analisar os efeitos de uma intervenção educativa à luz da Teoria da Aprendizagem Significativa sobre o conhecimento e a atitude de médicos e enfermeiros da atenção básica de saúde na avaliação do grau de incapacidade física na hanseníase.

**Método:**

Estudo de intervenção do tipo antes e depois, realizado com 122 profissionais, sendo 84 enfermeiros e 38 médicos, da Atenção Básica de Saúde de João Pessoa, Paraíba, em curso de capacitação sobre avaliação do grau de incapacidade física na hanseníase. Os dados foram coletados com instrumento próprio validado e analisados pelo teste qui-quadrado aderência e de proporção, com nível de significância de 5%.

**Resultados:**

Houve aumento dos escores de todos os itens do instrumento, com diferença estatisticamente significativa (p < 0,05) em 20 dos 32 itens, com destaque para os itens referentes à capacidade técnica do profissional para conduzir as etapas de anamnese, palpação dos nervos periféricos, avaliação sensitiva e motora. Destaca-se também que após a intervenção 5 itens obtiveram 100% de acertos.

**Conclusão:**

Intervenção educativa pautada na Teoria da Aprendizagem Significativa aperfeiçoou o conhecimento e a atitude dos profissionais de saúde na avaliação do grau de incapacidade física de pessoas com hanseníase.

## RESUMEN

**Objetivo:**

Analizar los efectos de una intervención educativa a la luz de la Teoría del Aprendizaje Significativo sobre el conocimiento y la actitud de los médicos y enfermeros de la Atención Primaria de la Salud en la evaluación del grado de discapacidad física en casos de lepra.

**Método:**

Estudio de intervención antes y después, realizado con 122 profesionales, 84 enfermeras y 38 médicos, de Atención Primaria de la Salud en João Pessoa, Paraíba, en un curso de capacitación sobre la evaluación del grado de discapacidad física en lepra. Los datos se recolectaron mediante un instrumento validado y se analizaron mediante la prueba de chi-cuadrado de adherencia y proporción, con nivel de significancia del 5%.

**Resultados:**

Hubo un aumento en las puntuaciones de todos los ítems del instrumento, con diferencia estadísticamente significativa (p < 0.05) en 20 de los 32 ítems, con énfasis en los ítems referentes a la capacidad técnica del profesional para realizar los pasos de la anamnesis, palpación de los nervios periféricos, evaluación sensorial y motora. Además, es de destacar que luego de la intervención, 5 ítems obtuvieron respuestas 100% correctas.

**Conclusión:**

La intervención educativa basada en la Teoría del Aprendizaje Significativo mejoró el conocimiento y la actitud de los profesionales de la salud en la evaluación del grado de discapacidad física de las personas con lepra.

## INTRODUCTION

Leprosy is a dermatoneurological disease that can cause physical disabilities in the face and in the upper and lower limbs, causing a series of problems, such as limitation to perform activities of daily living, reduced ability to perform work functions and restriction to participate in society, in addition to arousing stigma and prejudice^(1–2)^. Such disabilities can be classified in degrees ranging from 0, when the sensory and motor functions are preserved; 1, indicating changes in sensitivity and/or muscle strength; and 2, in the presence of visible deformities resulting from the disease, composing epidemiological indicators used for monitoring the disease^(3)^.

Despite the reduction in the number of cases of individuals with physical disabilities in the world scenario over the years, improvement in their management still represents a challenge for some countries, such as Brazil^(4)^, which holds 18.6% of all the recorded cases with disabilities in the world^(5)^. Of the 311,384 new cases recorded in the country between 2009 and 2018, 85,217 (27.4%) already had grade 1 or 2 disabilities at the time of diagnosis.

In view of this situation, the reduction of cases diagnosed with physical disability has been listed as a priority in the “2019–2022 National Strategy against Leprosy”, which requires professionals to perform early diagnosis, timely and appropriate treatment of cases and prevention of disabilities, so that cure of the disease is attained with minimal sequelae^(6–7)^.

Therefore, it is necessary to ensure that these disease control activities are developed in a decentralized and integrated way to Primary Health Care (PHC) services, from an assistance and comprehensive care network and, thus, ensure access to the diagnostic and therapeutic resources close to the user’s residence^(8–9)^.

Considering that, in order to provide care to people with leprosy, PHC professionals must be qualified, having adequate knowledge about the disease and expressing attitudes in accordance with the guidelines proposed by the Ministry of Health (*Ministério da Saúde*, MS), it is important to investigate which knowledge and attitudes PHC professionals have about physical disabilities, so that weaknesses, whenever detected, are resolved through educational interventions, in order to allow them to come to successful practices.

In this direction, the use of theories focused on learning and based on the previous knowledge of the target population subsidizes the planning and development of educational interventions that value the subject as an active participant in the construction of knowledge, as it is the case of Ausubel’s Meaningful Learning Theory (AMLT)^(10)^.

Thus, the proposal was to carry out an educational intervention on the assessment of the Degree of Physical Disability (DPD) of people with leprosy, according to the National Policy of Permanent Education in Health (*Política Nacional de Educação Permanente em Saúde*, PNEPS), which aims at qualifying health professionals based on the problems and difficulties arising from their work process^(11)^. Therefore, the goal was to analyze the effects of an educational intervention in the light of the Meaningful Learning Theory on the knowledge and attitude of Primary Health Care physicians and nurses in the assessment of the degree of physical disability in leprosy.

## METHOD

### Design of Study

An educational intervention study focused on the Knowledge and Attitude constructs about the assessment of the DPD in leprosy, with evaluation before and after, based on the AMLT^(10)^, with PHC physicians and nurses from the municipality of João Pessoa, Paraíba, Brazil.

To guide apprehension of the Knowledge and Attitude constructs, the following concepts were adopted: (a) Knowledge: it relates to the understanding of a given subject, to the recall of specific facts, within the educational system of which the individual is part; and (b) Attitude: it concerns the emotional dimension, referring to having opinions, feelings and beliefs, constantly, about a certain object, person or situation^(12)^.

### Population

The PHC of the municipality has 200 health teams distributed in five health districts, with a population of 392 professionals: 200 nurses and 192 physicians. To calculate the sample, a stratification procedure was performed considering a representative sampling plan of each health district, obtaining a sample of 119 professionals. In view of the possibility of sample losses in the course of the research, a percentage of 30% was increased in the number, totaling 155 professionals.

### Sample Selection and Definition Criteria

The following were established as inclusion criteria: being active during the data collection period and being available to participate in the intervention; and the exclusion criterion was having a participation frequency of less than 75%. The professionals were selected by the Coordination of the Leprosy Technical Area for convenience of the management to facilitate the care flow, recruited by the Health Managers of their respective Family Health Units (FHUs). 153 professionals started the intervention, with exclusion of those who were absent more than once (n = 31), accounting for a total of 122.

### Data Collection

The intervention, entitled “Training Course for Assessing the Degree of Physical Disability in Leprosy Patients”, was carried out with five groups from September to December 2019, with a workload of 20 hours, of which 16 were in-person, divided into 4 meetings, and 4 were devoted to reading texts and carrying out complementary activities.

**Chart 1:**
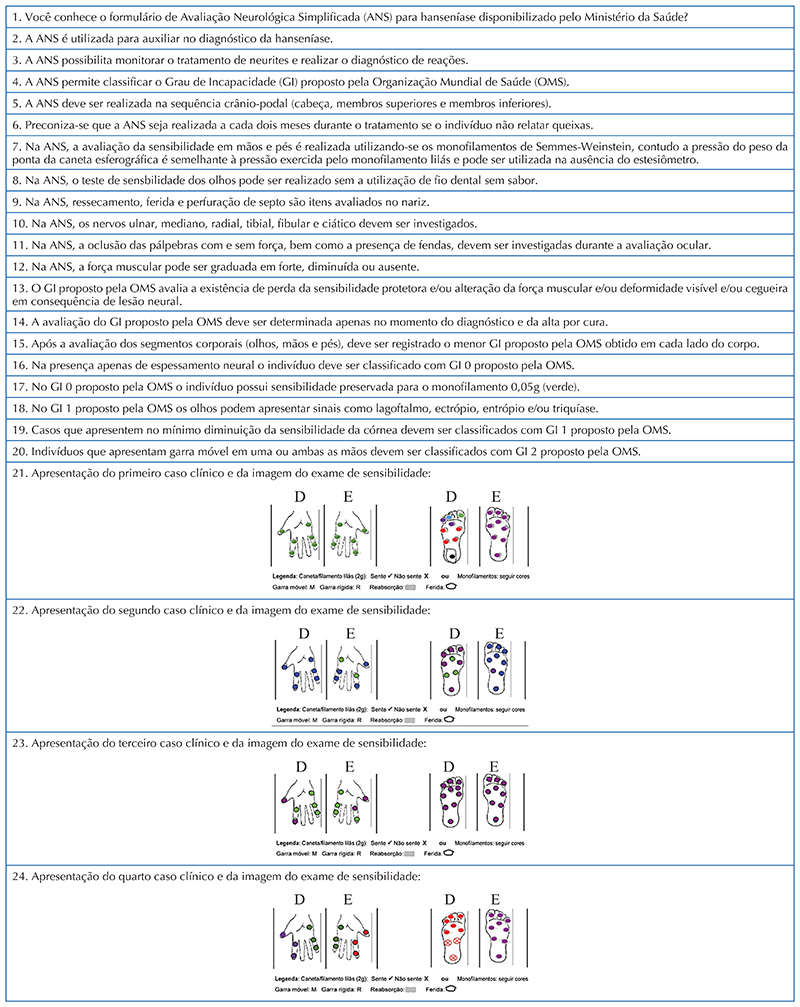
Items referring to the Knowledge construct of the instrument called “Knowledge and Attitude on the Assessment of the Degree of Physical Disability in Leprosy”. João Pessoa, Paraíba, Brazil, 2021. Source: Research data, 2021.

**Chart 2:**
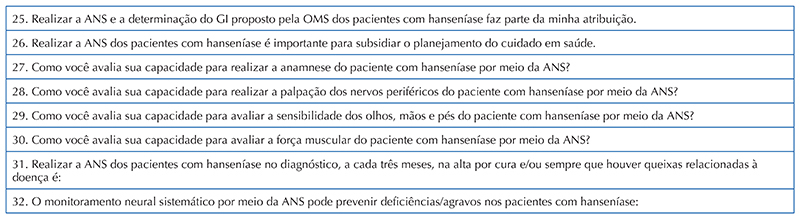
Items referring to the Knowledge construct of the instrument called “Knowledge and Attitude on the Assessment of the Degree of Physical Disability in Leprosy”. João Pessoa, Paraíba, Brazil, 2021. Source: Research data, 2021.

As Ausubel did not propose any rigorously systematized model for application of the theory, the stages^(13)^ elaborated from Ausubel’s guidelines for implementing MLT in teaching were adapted to operationalize the course, namely:

**–** 1^st^ stage: Quantitative assessment and presentation of the topic to be addressed, with prior application of the data collection instrument called “Knowledge and Attitude on the Assessment of the Degree of Physical Disability in Leprosy”, consisting of 32 questions, arranged as follows: 24 in the knowledge construct, subdivided into Simplified Neurological Assessment (SNA) (12) and Degree of Physical Disability (DPD) (12) and 8 in the attitude construct towards the DPD assessment in leprosy (Charts 1 and 2).

**–** 2^nd^ stage: Proposal of problem situations for the students to externalize their knowledge, through the opportunity of a moment of reflection on the practicalities and difficulties encountered to evaluate the DPD in PHC and discussion about the competences of the professionals in PHC to perform this evaluation.

**–** 3^rd^ stage: Proposal of problem situations to prepare the students for the presentation of knowledge, from the display of a clinical case of an individual diagnosed with leprosy and asking how to proceed with evaluation of the DPD, with generation of cognitive conflicts among the professionals by their interest to solve the problem.

**–** 4^th^ stage: Presentation of the knowledge, considering the principle of progressive differentiation, in which the general concepts must be addressed initially so that, progressively, they can be differentiated, discussing leprosy in general.

**–** 5^th^ stage: Continuation of the presentation of knowledge, based on dialog-related exposure moments about evaluation of the DPD and practical training of the SNA, taking into account the principle of integrative reconciliation to identify and explore inconsistencies between the theory and practice of PHC professionals. Theoretical and practical exercises and group discussions on clinical cases were also conducted.

**–** 6^th^ stage: Conclusion of the unit, with encouragement for discussion among the professionals about the strategies to put into practice the knowledge acquired during training.

**–** 7^th^ stage: Quantitative reassessment, with reapplication of the data collection instrument.

**–** 8^th^ stage: Closure of the course, through the participants› speech on positive and negative aspects, delivery of certificates and guidance manual produced by the researcher to assist in the evaluation of the DPD of people with leprosy in PHC.

### Data Analysis and Processing

The data were analyzed in the R statistical software, with application of descriptive statistical techniques, such as simple absolute and percentage frequencies for the categorical variables and organization of the results in tables. The chi-square adherence and proportion tests were applied, respectively, to verify adequacy of the probabilistic model to the research data and to verify possible differences in the professionals’ knowledge and attitude before and after the educational intervention, adopting a 5% significance level (p < 0.05).

### Ethical Aspects

The research followed the ethical principles set forth in Resolution No. 466/12 of the National Health Council and was approved by the Research Ethics Committee of the Center for Health Sciences at the Federal University of Paraíba, opinion No. 3,293,760. All the participants signed the Free and Informed Consent Form.

## RESULTS

There was predominance of females (87.7%), with a mean age of 43.8 years, undergraduate Nursing (68.9%) and in a private educational institution (51.6%), mean training time of 20.9 years, titration related to specialization (62.3%), belonging to Health District 1 (27.9%) and with more than 10 years of practice in the FHS (47.5%).

When asked about previous participation in leprosy training, 50% answered positively and 16.4% reported training in DPD evaluation. Regarding assistance, 66.4% stated that they never assisted people with leprosy.

Regarding the proportion of correct answers, it is observed that, after the intervention, there was an increase in all items of the instrument, which was statistically significant in 20 of the 32 (62.5%) questions. Before the intervention, no item had presented 100% of correct answers and, after the intervention, this rate of correct answers was obtained in 2 items of the knowledge construct and 3 of the attitude construct, namely: 1, 4, 26, 31 and 32.

Table 1 shows that in the SNA dimension of the Knowledge construct there was a statistically significant increase in the distribution of correct answers in 58.3% of the items, namely: knowledge of the form, purpose of use for diagnosis, periodicity of performance, sensitive evaluation of hands, feet and eyes, nasal evaluation and peripheral nerves, which deserves to be highlighted for having increased 76.2%.

**Table 1.** Distribution of the correct answers of the Knowledge construct related to the simplified neurological evaluation dimension before and after the educational intervention (n = 122). João Pessoa, Paraíba, Brazil, 2021.

Questions	Correct answers		p-value^a^
	Pre-intervention	Post-intervention	
	n (%)	n (%)	
1. Knowledge of the form	62 (50.8%)	122 (100%)	<0.001*
2. Purpose of use for diagnosis	12 (9.8%)	96 (78.7%)	<0.001*
3. Purpose of use in the treatment of neuritis	81 (66.4%)	118 (96.7%)	0.5540
4. Purpose of use to classify the DPD	90 (73.8%)	122 (100%)	0.6574
5. Achievement systematics	80 (65.6%)	118 (96.7%)	0.7540
6. Periodicity	24 (19.7%)	103 (84.4%)	<0.001*
7. Sensitive assessment of hands and feet	48 (39.3%)	116 (95.1%)	0.002*
8. Sensitive assessment of the eyes	22 (18.0%)	95 (77.9%)	<0.001*
9. Nasal evaluation	71 (58.2%)	111 (91.0%)	0.0405*
10. Peripheral nerves	6 (4.9%)	99 (81.1%)	0.0267*
11. Eye assessment	93 (76.2%)	120 (98.4%)	0.9330
12. Muscle strength graduation	91 (74.6%)	114 (93.4%)	0.8298

Significant result: (*) p-value < 0.05. ^a^ Proportion test. Source: Research data, 2021.

When analyzing the DPD dimension (Table 2), it is observed that there was a statistically significant increase in nine of the twelve items presented, which represents 75% of the total number of items. For two of the three items in which the proportion of correct answers did not show a significant increase after the intervention, there was an increase of nearly 50% in the correct answers. Additionally, the significant increase in the proportions of correct answers to the four clinical cases is highlighted, which reveals an improvement in attention and reflective ability to the characteristics of the cases and the positive effect of the intervention on knowledge regarding the DPD dimension.

**Table 2.** Distribution of the correct answers in the Knowledge construct regarding the Degree of Physical Disability dimension before and after the educational intervention (n = 122). João Pessoa, Paraíba, Brazil, 2021.

Questions	Correct answers		p-value^a^
	Pre-intervention	Post-intervention	
	n (%)	n (%)	
13. Purpose	87 (71.3%)	108 (88.5%)	0.2227
14. Periodicity	76 (62.3%)	115 (94.3%)	0.0297*
15. Recording	22 (18.0%)	101 (82.8%)	<0.001*
16. Classification (neural thickening)	31 (25.4%)	106 (86.9%)	0.0139*
17. Classification (sensitivity of the extremities)	40 (32.8%)	114 (93.4%)	0.7338
18. Classification (eye changes)	13 (10.7%)	103 (84.4%)	<0.001*
19. Classification (corneal sensitivity)	41 (33.6%)	108 (88.5%)	0.0375*
20. Classification (clawed hands)	60 (49.2%)	118 (96.7%)	0.4477
21. Clinical case 1	33 (27.0%)	98 (80.3%)	0.0002*
22. Clinical case 2	15 (12.3%)	104 (85.2%)	<0.001*
23. Clinical case 3	19 (15.6%)	107 (87.7%)	<0.001*
24. Clinical case 4	11 (9.0%)	97 (79.5%)	<0.001*

Significant result: (*) p-value < 0.05. ^a^ Proportion test. Source: Research data, 2021.

Regarding attitude, a construct distributed in Table 3, it is verified that there was an increase in the proportion of correct answers in 50% of the items evaluated. It is noteworthy that, in the others, where there was an increase in proportions, although not significantly, the rates of correct answers were already high, that is, above 70% before the intervention.

**Table 3.** Distribution of the correct answers for the Attitude construct before and after the educational intervention (n = 122). João Pessoa, Paraíba, Brazil, 2021.

Questions	Correct answers		p-value^a^
	Pre-intervention	Post-intervention	
	n (%)	n (%)	
25. Professional duty in carrying out the SNA and DPD	112 (91.8%)	121 (99.2%)	0.7879
26. Importance of the SNA	114 (93.4%)	122 (100%)	0.5819
27. Ability to perform anamnesis	7 (5.7%)	121 (99.2%)	<0.001*
28. Ability to palpate peripheral nerves	9 (7.4%)	107 (87.7%)	<0.001*
29. Ability to assess sensitivity	10 (8.2%)	120 (98.4%)	<0.001*
30. Ability to assess muscle strength	19 (15.6%)	120 (98.4%)	<0.001*
31. SNA periodicity	90 (73.8%)	122 (100%)	0.0607
32. SNA purpose	108 (88.5%)	122 (100%)	0.0807

Significant result: (*) p-value < 0.05. ^a^ Proportion test. SNA = Simplified Neurological Assessment. DPD = Degree of Physical Disability. Source: Research data, 2021.

It is also noted in Table 3 that, although before the intervention, more than 90% of the participants stated agreeing that performing the SNA and determining the DPD are part of their professional duties, less than 10% reported having the ability to perform them, with very different rates observed after the intervention.

## DISCUSSION

Although most of the study participants have been working in the FHS for more than 10 years (47.5%), which allows supposing that they know the problem involved in the care that should be provided to people with leprosy in PHC, only nearly half of the professionals (50.8%) knew the SNA form, indicated to assess integrity of the neural function and to determine the patients’ DPD, which goes against the national guidelines for the management and prevention of physical disabilities resulting from the disease^(14)^.

Although this percentage of professionals claimed to know the SNA form, it is verified that in the pre-intervention period, the frequency of correct answers regarding items such as peripheral nerves that should be investigated during the evaluation (4.9%), purpose of use of the form (9.8%), method of evaluating eye sensitivity (18%) and periodicity of SNA performance (19.7%) was less than expected.

After the intervention, all variables of the SNA dimension increased, noticing that the proportion of correct answers increased by 76.2% in the peripheral nerves item, 68.8% for purposes of use, 59.9% in the sensitive evaluation of the eyes and 64.7% in the periodicity with which the assessment should be carried out, resulting in significant changes in the professionals’ knowledge based on the educational intervention carried out.

The same can be observed for the moment to determine the DPD, in which, when clinical cases depicting signs and symptoms identified in people with leprosy were presented, frequencies of correct answers of only 27%, 12.3%, 15.6% and 9.0% were obtained in the pre-intervention for each of the 4 clinical cases, respectively.

Considering that these correct answer frequencies rose to 80.3%, 85.2%, 87.7% and 79.5% in the post-test, it is verified that, throughout the educational intervention, the professionals were able to develop clinical reasoning related to the disease, something fundamental to assist in the decision-making process and, thus, manage changes in the spaces in which they are inserted, enabling improvements in access, quality and humanization of the care provided to the population^(12)^. In addition to the favorable results for the knowledge construct, the relevance of the intervention can also be seen from the attitudes developed by the professionals, considering that more than 85% stated feeling capable of leading the step-by-step to carry out the SNA.

Even though, before the intervention, an expressive index of professionals believed that the SNA was part of their duties, that it supported the care planning and that it was necessary to carry it out periodically as recommended, the belief or feeling of inability to perform it among most of the participants stood as a barrier to possible future actions, even in the face of evidence of satisfactory knowledge. Attitudes like this can discourage the care of people with leprosy from the perspective of global and at the same time specialized assessment, as the attitudes adopted by professionals towards a decision process are a reflection of their beliefs and feelings, that is, of the what they believe and the feeling generated by this belief, which can exert both negative and positive influences on the care provided to people with leprosy.

The deficits in knowledge and attitude about aspects related to leprosy found in the pre-intervention can also be verified in other studies^(7,15–16)^, which reported presence of failures and inconsistencies between what is recommended by the MS and what is actually being carried out in PHC to control the isease in the country, which can reverberate in the emergence of complications due to the late inclusion of preventive measures.

In this context, and taking into account that only 16.4% of the professionals reported having participated in a specific course on DPD assessment in leprosy, which was even offered by the management, associated with evidence of dissociation between knowledge and attitudes already reported, the importance of expanding investments in professional training is highlighted, as well as for the managers to be concerned about offering educational strategies that address this issue, as the reduction in the number of new cases with physical disabilities is among the priorities of the disease coping strategies, both globally and in the national scope^(6,17)^.

Even given the reduced number of professionals with some specific training on the topic, access to information and experiences at different times and spaces configured the knowledge and attitudes about SNA and DPD preexisting in their cognitive structures, that is, the subsumers, which acted as anchors in the meaningful learning process^(10)^, providing incorporation of the information made available during the educational intervention to the professionals’ pre-existing cognitive structure.

With that, there were positive changes in the respondents’ conception, both in knowledge and in attitude, which contributed to empowerment in the decision-making process and in problem-solving and, therefore, to the improvement of the care that had been or will be be provided to people with leprosy in PHC in the municipality.

In accordance with the theoretical framework^(10)^, it is believed that these changes were facilitated by the use of previous organizers, which activated the subsumers already found in the professionals’ cognitive structure, but which were not being used. Previous organizers are introductory materials to be exposed to individuals before the actual material to be learned, such as the images, schemes and questions that were presented during their development, in order to act as “cognitive bridges” as they connected what the individuals already knew to what they should know.

In the literature, a number of studies support the importance of developing educational strategies for the qualification of professionals who work with leprosy. The Brazilian university developed a remote update course on disease control actions to qualify professionals in the State’s PHC, which allowed the teams to deepen their theoretical knowledge, as well as monitor their performance in disease elimination actions, consolidating the course as a viable educational tool to be used by managers from other locations^(18)^.

A study that investigated the contribution of training courses in leprosy offered to professionals evidenced that it was satisfactory to have promoted theoretical and practical knowledge and enabled participants to implement actions related to the disease in their health units, thus showing the importance of the periodic maintenance of courses of this nature to fight against the disease^(19)^.

On the other hand, a research study that evaluated the cognitive and attitudinal skills of PHC nurses in a hyperendemic Brazilian capital city showed that, even having participated in training on leprosy, 73.3% of the participants still did not feel qualified to treat patients, especially in terms of concerns regarding diagnostic suspicion, and that only 36.6% had specific training for the prevention of disabilities, which makes it difficult to implement measures to control the disease^(20)^.

A study carried out in the metropolitan region of Recife-PE also identified the effectiveness of training sessions carried out on leprosy as low, revealing the need to review their methodological structure based on the problematization of work, as recommended by the PNEPS, based on theory-practice integration, in order to improve the professionals’ performance with regard to early detection and timely treatment of cases^(21)^.

Given the heterogeneity of the disease in the country, the arguments about the results of educational interventions carried out in different contexts and the increase in the knowledge and attitude results in this study, we highlight the importance of not only carrying out training sessions, but planning them based on anchoring in a theoretical framework that takes into account the previous constructions of the target audience, so that they can be truly effective and reflect in transformations that materialize in different practice scenarios, allowing for a glimpse of promising results for improving health care quality, particularly in the context of prevention and care actions in leprosy.

## CONCLUSION

The educational intervention on DPD assessment in leprosy improved the knowledge and attitude of the PHC professionals, with an increase in the scores of all items in the questionnaire at the post-intervention moment; it can be inferred that significant learning occurred from the interaction between the ideas that were presented in the intervention with the pre-existing ones in their cognitive structures. Given the positive effects of the educational intervention based on the AMLT, it is suggested to offer periodic training sessions that value the professionals’ prior knowledge and attitudes and their active participation in the teaching-learning process, in order to promote retention of what was learned and to advance in control of the disease and its repercussions.

Although the objective directed to the effects produced in the knowledge and attitude constructs has been successfully achieved, the duration of the concise training course is pointed out as a limitation of this study to minimize the number of absences of professionals from the FHUs, limiting practical training and non-observation of the implementation of the knowledge acquired in the professionals’ routine, that is, advancement of the study into the practice.

## SUPPLEMENTARY MATERIAL

The following online material is available for this article.

Link to access the complete thesis: https://repositorio.ufpb.br/jspui/handle/123456789/21030. Additional data on demand.
